# Genetic Polymorphisms in *LDLR*, *APOB*, *PCSK9* and Other Lipid Related Genes Associated with Familial Hypercholesterolemia in Malaysia

**DOI:** 10.1371/journal.pone.0060729

**Published:** 2013-04-08

**Authors:** Say-Hean Lye, Jagdish Kaur Chahil, Pramod Bagali, Livy Alex, Jamunarani Vadivelu, Wan Azman Wan Ahmad, Siew-Pheng Chan, Meow-Keong Thong, Shamsul Mohd Zain, Rosmawati Mohamed

**Affiliations:** 1 INFOVALLEY® Group of Companies, Jalan Tasik, MINES Resort City, Selangor, Malaysia; 2 Faculty of Medicine, University of Malaya, Kuala Lumpur, Malaysia; 3 The Pharmacogenomics Laboratory, Department of Pharmacology, Faculty of Medicine, University of Malaya, Kuala Lumpur, Malaysia; University of Bonn, Institut of Experimental Hematology and Transfusion Medicine, Germany

## Abstract

Familial hypercholesterolemia (FH) is an autosomal dominant disorder characterized by elevations in total cholesterol (TC) and low density lipoprotein cholesterol (LDLc). Development of FH can result in the increase of risk for premature cardiovascular diseases (CVD). FH is primarily caused by genetic variations in *Low Density Lipoprotein Receptor* (*LDLR*), *Apolipoprotein B* (*APOB*) or *Proprotein Convertase Subtilisin/Kexin type 9* (*PCSK9*) genes. Although FH has been extensively studied in the Caucasian population, there are limited reports of FH mutations in the Asian population. We investigated the association of previously reported genetic variants that are involved in lipid regulation in our study cohort. A total of 1536 polymorphisms previously implicated in FH were evaluated in 141 consecutive patients with clinical FH (defined by the Dutch Lipid Clinic Network criteria) and 111 unrelated control subjects without FH using high throughput microarray genotyping platform. Fourteen Single Nucleotide Polymorphisms (SNPs) were found to be significantly associated with FH, eleven with increased FH risk and three with decreased FH risk. Of the eleven SNPs associated with an increased risk of FH, only one SNP was found in the *LDLR* gene, seven in the *APOB* gene and three in the *PCSK9* gene. SNP rs12720762 in *APOB* gene is associated with the highest risk of FH (odds ratio 14.78, p<0.001). Amongst the FH cases, 108 out of 141 (76.60%) have had at least one significant risk-associated SNP. Our study adds new information and knowledge on the genetic polymorphisms amongst Asians with FH, which may serve as potential markers in risk prediction and disease management.

## Introduction

Familial hypercholesterolemia (FH) (ICD-10 code E78.0) was the first genetic disease of lipid metabolism to be clinically and molecularly characterized [1]. It is an inherited disorder of lipoprotein metabolism, transmitted in an autosomal dominant manner [2]. FH is characterized by elevated levels of low density lipoprotein cholesterol (LDLc) and total cholesterol (TC) in the circulation, deposits of cholesterol in peripheral tissues, presence of tendon xanthomas and accelerated atherosclerosis, leading to premature cardiovascular events [1], [3], [4], [5], [6].

Heterozygous FH is one of the most frequent Mendelian disorders with a frequency of 1 in 500 but has a much higher incidence in certain populations, such as the Afrikaners, Christian Lebanese, Finns, and French-Canadians [7]. The frequency of homozygous FH is 1 in a million, with symptoms appearing in childhood [8].

FH can result primarily from mutations in either *Low Density Lipoprotein-Receptor* gene *(LDLR)*, *Apolipoprotein B-100* gene (*APOB*), *or Proprotein Convertase Subtilisin/Kexin type 9* gene (*PCSK9*), singly or in combination [1]. Genetic variations in the *LDLR* gene are commonly loss-of-function mutations, which result in increased plasma LDLc levels [9]. Genetic variations in the *APOB gene*, and the *PCSK9* gene give rise to the same lipid homeostasis functional defects [1]. To date, genetic variants in an excess of over 1000 have been identified in the *LDLR, APOB & PCSK9* gene as reported by British Heart Foundation (BHF) and other public databases. In addition, other genes associated with lipid control and regulatory regions such as *Upstream Transcription Factor 1* gene (*USF1*), Apolipoprotein *E* gene (*APOE*), *Lipoprotein Lipase* gene (*LPL*), *Fibrinogen Beta Chain* gene (*FGB*), and *Hepatic Lipase* gene (*LIPC*) can manifest as hypercholesterolemia and have been shown to predispose to premature cardiovascular diseases [10], [11], [12], [13], [14]. Results from SNPs study should be interpreted with caution, as SNPs may exert their effects individually, or multiple SNPs can act synergistically to cause a functional difference between haplotypes. Interaction among multiple SNPs may jointly affect a disease’s risk. Assessing the independent individual SNP without considering the SNP-SNP interactions forms (even on SNPs that show very weak associations with estimated odds ratios) will fail to discover weak associations [15], [16] .

Clinical management of FH focuses on early detection and control of hypercholesterolemia to decrease the risk of atherosclerosis and to prevent premature cardiovascular disease [17]. Establishing an accurate diagnosis of FH is often difficult. In spite of its prevalence, and considerable benefit associated with its early detection and treatment, FH is often under-diagnosed in many countries [18], [19]. Systematic genetic screening for mutations in those at risk of FH has been found to be cost effective and will help in better prognosis [20]. However, most genetic studies in FH were conducted in non-Asian populations and the allelic variants prevailing here in South East Asia are not known. In South East Asia, specifically in Malaysia, only few studies have been carried out on these genes [21], [22], [23], [24], [25], [26]. Thus, the aim of this study is to determine the genetic variants in the *LDLR*, *APOB*, *PCSK9* and other lipid related genes in a study cohort with clinical FH.

## Materials and Methods

### Subject Recruitment

Consecutive 141 patients with high LDLc levels above 4 mmol/L were recruited between January 2007 and September 2009 from the medical out-patient clinics at the University Malaya Medical Centre (UMMC), Kuala Lumpur. The FH-Dutch Lipid Clinic Network (DLCN) criteria [27], [28] was adopted as the diagnostic scoring method to clinically diagnose/screen for FH, excluding molecular diagnosis criterion, and stratify subjects into possible FH, probable FH or definite FH. One hundred and one control subjects consisted of those who were genetically unrelated, with normal LDLc levels, absence of family history of FH, hyper/hypothyroidism, chronic kidney disease, diabetes, chronic liver disease and characterized as not FH by DLCN criteria (Table S1). The protocol was approved by the UMMC’s Medical Ethics Committee (Ref: 546.16) and written informed consents were obtained from all patients.

### Questionnaire and Data Collection

The data included socio-demographic characteristics (age, sex, and occupation), personal and family history of hypertension, hypercholesterolemia, CVD and other lifestyle habits, such as smoking status, and physical activity. Body mass index (BMI), waist circumferences (WC) and blood pressure were also measured.

### DNA Isolation

Genomic DNA from all subjects was isolated from whole blood using QIAamp DNA Mini Kit (QIAGEN, USA) in 200 µl of total volume according to user protocol. Qualitative and quantitative estimations were carried out on the DNA samples. All DNA samples were normalized to concentration of 50 ng/µl for genotyping.

### Selection of Genes/SNPs and Microarray Probes Synthesis

Genetic variations implicated in FH from three publically available databases, BHF (www.ucl.ac.uk) [29], dbSNP (ncbi.nlm.nih.gov/SNP/) [30] and SNPedia (www.snpedia.com), were selected based on the following attributes: i) conventional SNPs known to cause FH in genes encoding *LDLR*, *APOB* and *PCSK9* ii) SNPs in *USF1*, *APOE*, *LPL*, *FGB* and *LIPC* that were known to have functional effects by in vitro assays or were non-synonymous in lipid regulatory regions. Though our initial research and mining led us to 1850 SNPs, which were sent to Illumina for designing the probes, only 1536 could be of designable standards as per Illumina criteria.

A tool called Assay Design Tool (ADT) of Illumina ranks SNPs based on an in-built algorithm where SNPs scoring below 0.4 have a rank of zero suggesting the probe is not designable by Illumina. SNPs scoring between 0.4 and 0.6 get a rank 0.5 whereas a score above 0.6 is ranked 1. SNPs scoring 0.5 and 1 are technically ranked as SNPs that can be successfully designed as probes by Illumina. The assay has an average 30-fold redundancy for each probe thus making the quality control robust. In all, 231 probes had score of 0.5 and 1305 had a score of 1.0. Only 1536 SNPs were chosen because that was the maximum plexity Illumina platform could accommodate.

Most of the reported studies till date were from Caucasian population, and hence we were keenly interested to re-look at the reported SNPs using a population based approach, and check if the interpretations are extrapolatable to Asian population.

Designability scores were graded and qualified probes were selected and synthesized for the custom GoldenGate™ genotyping assay (GGGT) (www.illumina.com). Of the 1850 SNPs mined, 1536 SNPs were synthesized as probes, comprising of 811 in *LDLR*, 245 in *APOB*, 284 in *PCSK9*, and another 196 lipid-regulatory related SNPs.

### Genotyping

Genotyping was performed on Universal BeadChips (Illumina, USA) according to the manufacturer’s protocol and was carried out in compliance with MIAME (Minimum Information about a Microarray Experiment) guidelines [31]. All the raw data from our GGGT microarray assays were imported into the GenomeStudio™ software (Illumina, USA) for allelic analysis and deviation from Hardy-Weinberg equilibrium. Average call rate of 70–80% was observed, which is expected of a custom GGGT assay.

### Statistical Analysis

Statistical analysis was performed using the SPSS software v16.0 (SPSS Inc., Chicago, Illinois). The test of normality (Kolmogorov-Smirnov) was employed to determine the normality of the variables. Descriptive analysis and statistical significance of the association were assessed by independent t-test. Logistic regression was applied to obtain the Odds Ratio (OR) and the p-values for the tested SNPs (p-values ≤0.05 were considered to be significant). An OR>1.0 was used as the cut-off for the baseline of risk-associated SNPs, and the baseline risk-lowering SNPs as OR<1.0. An OR equal to 1 was a neutral value and deemed as normal. Analysis of variance (ANOVA) test was conducted for comparison of means between clinical profiles and three genotype groups. Minor allele frequency (MAF) for this study was calculated. MAF for the most closely related ethnic group to our study were also extracted from public database (NCBI dbSNP Build 137). A Chi-Square Test was performed to determine whether there was a significant difference between our study’s MAF and public databases’ MAF. Bonferroni correction for multiple comparisons of SNPs on the same gene was performed. Unless otherwise specified, all data were presented as means and standard deviations.

### Additional Validation of Genotype Calls by Sequencing

Microarray calls were validated by blindly re-genotyping some SNPs in a number of subjects randomly selected from cases using DNA sequencing. Primers were synthesized for regions encompassing a few significant SNPs and the PCR products amplified from the genomic DNA of FH cases were sent for sequencing (First BASE Laboratories, www.base-asia.com).

## Results

### Subjects Demographics and Clinical Profiles

Of the 141 FH subjects and 111 control subjects included in the study, 24 were classified as definite FH, 25 as probable FH and 92 as possible FH from cases based on DLCN criteria. There was no significant gender bias observed between FH subjects (73 male: 68 female) and control subjects (46 male: 65 female) that were recruited into the study (p = 0.104). The mean age of the FH subjects recruited was 46.84 (SD ±11.2), while control subjects were 40.00 (SD ±9.3) with significant difference (p<0.001). The BMI (p = 0.239), waist circumference (p = 0.356) and HDL cholesterol (HDLc) level (p = 0.420) were similar between the two study groups. FH subjects were found to have significantly higher levels of triglycerides (TG) (p = 0.001), TC and LDLc (p<0.001) as compared to controls (Table 1).

**Table 1:** Demographics and clinical profiles of the subjects.

Characteristic	Over all FH cases	Definite FH	Probable FH	Possible FH	Controls	p-value Over all FH cases vs. Controls
Males : Females	73∶68	11∶13	15∶10	47∶45	46∶65	0.104
Age (years)	46.84 (±11.2)	42.37 (±17.4)	45.60 (±18.6)	48.34 (±8.5)	40.00 (±9.3)	<0.001
BMI (kg/m^2^)	26.42 (±5.4)	22.79 (±5.1)	26.49 (±5.2)	27.32 (±5.2)	25.62 (±4.9)	0.239
WC (cm)	86.21 (±17.5)	76.64 (±19.1)	85.37 (±18.6)	88.90 (±16.0)	83.05 (±12.2)	0.356
TG (mmol/L)	1.79 (±1.0)	1.99 (±1.8)	2.00 (±1.0)	1.69 (±0.7)	1.23 (±0.7)	0.001
TC (mmol/L)	8.86 (±5.1)	13.23 (±10.7)	9.51 (±2.4)	7.47 (±1.2)	5.18 (±0.9)	<0.001
HDLc (mmol/L)	1.25 (±0.7)	1.17 (±0.4)	1.53 (±1.5)	1.20 (±0.3)	1.34 (±0.3)	0.420
LDLc (mmol/L)	6.37 (±2.3)	9.23 (±3.4)	6.96 (±2.3)	5.49 (±1.0)	3.28 (±0.7)	<0.001

BMI, Body Mass Index.

WC, Waist circumference.

TG, Triglyceride.

TC, Total Cholesterol.

HDLc, High Density Lipoprotein Cholesterol.

LDLc, Low Density Lipoprotein Cholesterol.

The data are expressed as mean (±SD).

p-values were obtained by comparing the phenotypes between the two groups using Student’s t-test.

### FH Associated SNPs

A total of 14 SNPs were found to be significantly associated with FH. Eleven out of 14 were associated with high risk of FH (OR >1), while the remaining three were protective against FH (OR <1). Of the 11 associated SNPs, one (rs2569556) was found in the *LDLR* gene, seven in the *APOB gene* (rs12720762, rs13306187, rs13306194, rs12714238, rs12720772, rs57825321 and rs41291161) and three (rs12084215, rs565436 and rs28362269) in the *PCKS9* gene. The *APOB* rs12720762 is associated with the highest risk of FH with OR of 14.78. Amongst the FH subjects, 108 (76.60%) subjects have had at least one significant risk-associated SNP.17 out of 24 definite FH subjects (70.83%), 19 out 25 probable FH subjects (76%) and 72 out of 92 possible FH subjects (78.26%) had at least one significant risk-associated SNP (Table 2).

**Table 2:** FH associated SNPs, (p<0.05).

Gene	rs number	Nucleotidechange	p-value	OR (CI)
*LDLR*	rs2569556	[G>A]	0.0140	1.77 (1.12–2.78)
*APOB*	rs13306187	[G>A]	<0.0001	6.76 (3.28–13.90)
*APOB*	rs13306194	[G>A]	0.0154	2.25 (1.17–4.34)
*APOB*	rs12714238	[G>A]	<0.001	8.04 (3.20–20.20)
*APOB*	rs12720772	[G>A]	0.0130	2.00 (1.16–3.46)
*APOB*	rs12720762	[G>C]	<0.001	14.78 (5.03–43.44)
*APOB*	rs41291161	[T>A]	<0.0001	11.51 (4.32–30.69)
*APOB*	rs57825321	[A>T]	0.0304	2.02 (1.07–3.83)
*APOB*	rs12714254	[T>G]	<0.001	0.22 (0.11–0.50)
*PCSK9*	rs12084215	[C>A]	0.0064	3.87 (1.46–10.23)
*PCSK9*	rs565436	[A>G]	0.0020	5.00 (1.80–13.89)
*PCSK9*	rs28362269	[G>A]	<0.001	5.43 (2.76–10.65)
*USF1*	rs3737787	[G>A]	0.0174	0.55 (0.33–0.90)
*USF1*	rs2516839	[G>A]	0.0317	0.67 (0.46–0.97)

rs number, NCBI Reference SNP (rs) Number, an identification tag assigned by NCBI to SNPs [30].

CI, Confidence interval.

Odds ratio (OR) between groups was determined by logistic regression.

The *APOB* rs57825321 and *USF1* rs3737787 and rs2516839 were found to have a protective effect against FH in this case-control association study (Table 2). No significant associations were found with the other 1522 SNPs (99.09%) genotyped (Statistical data not shown but available upon request).

### SNPs Association with Clinical and Demographic Profile

We also investigated the association of significant SNPs with the clinical and demographic profile of FH patients. There was no significant association between most of the SNPs with the clinical and demographic profile. However, the *APOB* rs13306194 and rs57825321 were significantly associated with HDLc level (p<0.001). *APOB rs12720772* was associated with TC (p = 0.0275) and BMI (p = 0.0337) while the *PCSK9* rs12084215 was associated with HDLc level (p = 0.0090) and BMI (p = 0.0228) (Table 3, 4, 5, and 6). Clinical and demographic profile of control subjects was also examined similarly. No significant association was seen between most of the SNPs with the clinical and demographic profile. However, rs12084215 (*PCSK9*) was significantly associated with TG level (p = 0.0052) and waist circumference measurement (p = 0.0214) (Table 7).

**Table 3:** Comparison of clinical profiles between rs13306194 genotypes among FH patients.

Clinical Profiles	GG (% = 88)	GA (% = 12)	AA (% = 0)	p-value
Age	47.11±(10.9)	46.18±(12.3)	–	0.7463
TG	1.83±(1.1)	1.54±(0.7)	–	0.2905
TC	8.89±(5.4)	8.86±(2.7)	–	0.9848
HDLc	1.15±(0.3)	1.94±(1.8)	–	<0.001
LDLc	6.42±(2.4)	6.16±(2.4)	–	0.6622
BMI	26.44±(5.5)	26.29±(5.5)	–	0.9173
WC	86.57±(17.3)	82.38±(19.8)	–	0.3720

Data are presented in mean ± SD.

p-values were obtained by comparing the phenotypes among the genotypes using Analysis of Variance (ANOVA).

**Table 4:** Comparison of clinical profiles between rs57825321 genotypes among FH patients.

Clinical Profiles	AA (% = 86)	AT (% = 14)	TT (% = 0)	p-value
Age	47.02±(11.2)	45.95±(12.0)	–	0.6965
TG	1.83±(1.1)	1.53±(0.7)	–	0.2456
TC	8.89±(5.5)	8.76±(2.5)	–	0.9169
HDLc	1.15±(0.3)	1.88±(1.6)	–	<0.001
LDLc	6.43±(2.4)	6.13±(2.2)	–	0.5995
BMI	26.51±(5.5)	25.97±(5.1)	–	0.6817
WC	86.79±(17.4)	82.44±(18.6)	–	0.3310

Data are presented in mean ± SD.

p-values were obtained by comparing the phenotypes among the genotypes using Analysis of Variance (ANOVA).

**Table 5:** Comparison of clinical profiles between rs12720772 genotypes among FH patients.

Clinical Profiles	GG (% = 48)	GA (% = 52)	AA (% = 0)	p-value
Age	46.69±(9.0)	47.39±(12.0)	–	0.7175
TG	1.63±(0.8)	1.80±(0.8)	–	0.2683
TC	7.89±(1.9)	8.88±(2.8)	–	0.0275
HDLc	1.23±(0.5)	1.30±(0.9)	–	0.6228
LDLc	5.89±(1.6)	6.68±(2.8)	–	0.0614
BMI	25.18±(3.5)	27.10±(5.9)	–	0.0337
WC	85.04±(13.5)	86.11±(18.8)	–	0.7290

Data are presented in mean ± SD.

p-values were obtained by comparing the phenotypes among the genotypes using Analysis of Variance (ANOVA).

**Table 6:** Comparison of clinical profiles between rs12084215 genotypes among FH patients.

Clinical Profiles	CC (% = 91)	CA (% = 9)	AA (% = 0)	p-value
Age	44.39±(11.3)	53.50±(2.6)	–	0.0558
TG	1.72±(0.8)	1.68±(0.9)	–	0.9069
TC	8.35±(3.1)	7.65±(1.4)	–	0.5927
HDLc	1.18±(0.5)	2.32±(2.8)	–	0.0090
LDLc	6.32±(3.0)	4.56±(1.8)	–	0.1602
BMI	25.80±(5.7)	31.57±(5.9)	–	0.0228
WC	84.33±(17.0)	94.58±(6.4)	–	0.1518

Data are presented in mean ± SD.

p-values were obtained by comparing the phenotypes among the genotypes using Analysis of Variance (ANOVA).

**Table 7:** Comparison of clinical profiles between rs12084215 genotypes among Control subjects.

Clinical Profiles	CC (n = 71%)	AC (n = 26%)	AA (n = 3%)	p-value
Age	38.05±(9.11)	37.67±(5.92)	41.00±(4.24)	0.8686
TG	1.07±(0.57)	2.90±(n/a)	–	0.0052
TC	5.11±(0.93)	6.20±(n/a)	–	0.2659
HDLc	1.42±(0.35)	0.98±(n/a)	–	0.2368
LDLc	3.20±(0.78)	3.90±(n/a)	–	0.3934
BMI	25.55±(4.91)	24.98±(5.08)	25.14±(2.27)	0.9313
WC	78.64±(8.96)	98.50±(14.85)	–	0.0214

Data are presented in mean ± SD.

p-values were obtained by comparing the phenotypes among the genotypes using Analysis of Variance (ANOVA).

### Minor Allele Frequency

Information regarding gene, rs number, SNPs region, chromosome, nucleotide change and allele frequency of the associated SNPs observed from this study was summarized. Although, in our study the minor allele of the significant SNPs matched with the information in public database, six of the SNPs (rs13306187 MAF = 0.111, rs12714238 MAF = 0.072, rs12720772 MAF = 0.301, rs41291161 MAF = 0.077, rs57825321 MAF = 0.403, and rs12714254 MAF = 0.405) differed in frequency (p<0.05) (Table 8).

**Table 8:** Minor allele frequency of significant SNPs.

Gene	rs no.	Region	Chr	Nucleotide change	MAF (Total)	MAF (Cases )	MAF (Controls)	MAF (PD)	p-value(Total vs. PD)	MAF source
LDLR	rs2569556	Intron 6	19	G>A	0.263	0.316	0.223	0.209	0.3853	HapMap-HCB
APOB	rs13306187	Exon 25	2	G>A	0.111	0.197	0.043	0.035	0.0314	HapMap-HCB
APOB	rs13306194	Exon 12	2	G>A	0.091	0.128	0.061	0.133	0.2784	Pilot 1 CHB+JPT low coverage panel
APOB	rs12714238	Intron 5	2	G>A	0.072	0.136	0.022	0.011	0.0311	Pharmacogenetics Network for Cardiovascular Risk Therapy
APOB	rs12720772	Intron 18	2	G>A	0.301	0.352	0.260	0.000	<0.001	HapMap-CHB
APOB	rs12720762	Intron 1	2	G>C	0.076	0.159	0.014	0.021	0.0741	Pharmacogenetics Network for Cardiovascular Risk Therapy
APOB	rs41291161	Intron 14	2	T>A	0.077	0.149	0.021	0.001	<0.001	ABECASIS CLINICAL PANEL
APOB	rs57825321	Intron 16	2	A>T	0.403	0.429	0.369	0.158	<0.001	Pilot 1 CHB+JPT low coverage panel
APOB	rs12714254	Intron 3	2	T>G	0.405	0.342	0.454	0.100	<0.001	Pilot 1 CHB+JPT low coverage panel
PCSK9	rs12084215	Intron 3	1	C>A	0.102	0.164	0.046	NA	NA	NA
PCSK9	rs565436	Intron 9	1	A>G	0.079	0.145	0.036	0.100	0.6627	Pilot 1 CHB+JPT low coverage panel
PCSK9	rs28362269	Intron 9	1	G>A	0.112	0.188	0.053	0.059	0.1289	Pilot 1 YRI low coverage panel
USF1	rs2516839	5′ UTR	1	G>A	0.408	0.360	0.447	0.366	0.5197	HapMap-CHB
USF1	rs3737787	3′ UTR	1	G>A	0.163	0.117	0.199	0.250	0.1032	HapMap-HCB

rs no, NCBI Reference SNP (rs) Number, an identification tag assigned by NCBI to SNPs.

Chr, Chromosome.

p-value obtained by comparing frequencies using Chi-Square Test.

MAF (Total), minor allele frequency obtained from total sum of case and control subjects in this study.

MAF (PD), minor allele frequency information from public database, NCBI dbSNP Build 137.

### Bonferroni Correction

Five out of the eight significant SNPs of *APOB* gene (rs13306187, rs12714238, rs12720762, rs41291161, rs12714254) and all three *PCSK9* gene SNPs (rs12084215, rs565436 and rs28362269) survived significance after a conservative Bonferroni correction for multiple testing, (0.05/9, p<0.0056) and (0.05/4, p<0.0125) respectively. However, none of the SNPs of *USF1* gene is significant following Bonferroni correction. These 8 SNPs are thus of sufficient interest to warrant further investigation.

### Additional Validation of Genotype Calls by Sequencing

A few samples were sent for sequencing to rule out any genotyping errors and results were concordant with the genotype calls generated from the microarray data (Figure S1).

## Discussion

Fourteen out of 1536 SNPs evaluated in this study were significantly associated with FH, with 11 SNPs associated with increasing risk for FH, while the remaining three SNPs associated with decreasing risk for FH. Among the risk-increasing SNPs, allele A of rs2569556 in *LDLR* gene was identified among 55 out of 141 FH cases (39.0%), with 48 heterozygous and seven in homozygous genotypes. SNP rs13306187 in *APOB* gene also demonstrated risk association on allele A among 11 FH cases. Ten of the FH cases were heterozygous and only one of the FH case was observed in homozygous genotype. Other risk associated SNPs on *APOB* gene were only observed to occur in heterozygous genotype and these included 64 cases for rs12720772; six cases for rs41291161; 121 cases for rs57825321; 17 cases for rs13306194, six cases for rs12714238, and four cases for rs12720762. Three *PCSK9* SNPs were observed to be associated with increased risk in a heterozygous manner (six cases for rs565436, six cases for rs12084215, and 15 cases for rs28362269). Only two risk-elevating SNPs (rs2569556 in *LDLR* gene and rs13306187 in *APOB* gene) were observed to be in the homozygous state; while other 9 SNPs presented in a heterozygous manner, which may demonstrate a milder phenotypic effect of FH. In general, 17 definite FH subjects (70.83%), 19 probable FH subjects (76%) and 72 possible FH subjects (78.26%) had at least one SNP out of the risk-increasing SNPs. The remaining 33 FH subjects (23.40%) did not have any risk-increasing SNPs indicating that there are other genetic or environmental factors causing hypercholesterolemia that were undetected by our study and which has the potential for future investigation. Besides the risk-increasing SNPs, there were 3 other SNPs with OR<1 (rs12714254 in *APOB* gene, and rs2516839 and rs3737787 in the *USF1* gene) which confer lower risk against FH. *USF1* gene was studied because *USF1* protein regulates the transcriptional activation of a variety of genes involved in glucose, lipid and apolipoproteins (*APOCIII*, *APOAII* and *APOE*) metabolism in the development of atherosclerosis [32], [33], [34], [35]. Results for SNPs in other candidate genes such as *APOE, LPL, FGB* and *LIPC* were analysed and found not to be significant in our study (p>0.05).

Clinical parameters were compared between the significant genotypes among FH patients (Table 3, 4, 5, and 6). Allele A of *APOB* rs12720772 in heterozygous GA patients is associated with significantly higher level of plasma TC compared to the G allele (p = 0.0275), while allele A in *APOB* rs12720772 (p = 0.0337) and *PCSK9* rs12084215 (p = 0.0228) were associated with higher BMI. Interestingly, we observed that 3 risk alleles have the effect of significantly higher level of plasma HDLc in their heterozygous form in 3 SNPs (GA, rs13306194; AT, rs57825321 of *APOB*; CA, rs12084215 of *PCSK9*). For control subjects, similar approach for clinical and demographic profile was also performed. Only allele A in SNP rs12084215 of *PCSK9* was associated with higher level of TG (p = 0.0052) and waist circumference measurement (p = 0.0214) with heterozygous AC (Table 7). It is noteworthy that all the parameter-associated SNPs presented with homozygous wild type (+/+) and heterozygous (+/−) but extremely low or no homozygous mutant (−/−). This finding is commonly observed among the heterozygous FH associated SNPs [36] (Table 3, 4, 5, 6, and 7).

MAF from the studied population were calculated and compared with the MAF information on public database. Han Chinese subjects, where available, were selected as the targeted group for comparison as this Asian ethnicity were believed to closely resemble the ethnic groups of our study population. The results demonstrated that six of our SNPs differed in the frequency of MAF in public database [37]. Disparity in frequency could be due to founder-effects, natural selection or multi-ethnic groups in the study population [7], [38] (Table 8).

Out of the 1536 SNPs that were studied, 1522 SNPs (99.09%) did not show any significant result or association. This is because besides the SNPs being mono-allelic (non-polymorphic) [37], we also observed an almost equal number of the predicted risk alleles present in both case subjects and control subjects across our 252 samples. Thus analyses of these polymorphisms were not statistically significant and therefore, regarded as having non-pathogenic phenotype. These findings suggest that many SNPs published in public databases might just be non-pathogenic polymorphisms in Malaysia. This will require further validation in a larger population of Asian descent.

For the SNPs association study, we included all significant SNPs, inclusive of SNPs with relatively low odds ratio (*LDLR* rs2569556, OR 1.77), as SNPs usually work with other functionally relevant SNPs additively or synergistically, to manifest a disease condition in certain population [16]. Therefore, including these SNPs with relatively low ORs might aid future research on SNP-SNP interaction and polygenic effect of FH. Furthermore, 12 SNPs that were reported as significant were in the intronic and untranslated region (UTR) of genes. These SNPs could be in linkage disequilibrium with other functional SNPs involved in potential regulatory regions or splice site variants that may be associated with lipid related disorders. Exons 2 to 6 fall in the ligand binding region while exons 7 to 14 coded for epidermal growth factor precursor (EGFP)-like domain in *LDLR* gene. Although SNP rs2569556 is in intron 6, this polymorphism may contribute to the disturbance in ligand binding or the internalization of the receptor into the liver cell [29], [39].

SNPs rs2569556, rs12720772, rs57825321 and rs12714254 were found to deviate from HWE during the post-hoc analysis (Table S2). These SNPs were retained because not necessarily all HWE deviations indicate genotyping errors. SNPs deviating from HWE may confound trait-allele association as they are thought to reflect genotyping error [40], [41] although the contrary has also been argued [42]. Presence of a common deletion polymorphism, a mutant PCR-primer site or a tendency to miscall heterozygotes as homozygotes could also lead to deviation [43]. Deviation from HWE, assuming sources of error have been eliminated, may indicate the association of a locus with disease. As FH homozygous is so rare (one in a million) and deleterious, low count or complete absence of homozygous allele deviated significantly from HWE.

There are several limitations in the current clinical setup on how a subject is diagnosed as FH based solely on elevated cholesterol levels, physical manifestations and family history. Hypercholesterolemia is a silent disease and the underlying aetiology of FH is not routinely defined. Without proper genetic screening, “over-diagnosis” or misdiagnosis of FH may take place because the condition might overlap with other forms of dyslipidemia, sedentary lifestyle, inappropriate dietary intake or metabolic syndrome, which would contribute to hypercholesterolemia. Similarly, FH subjects with milder phenotypes who maintain a healthy lifestyle may have normal cholesterol levels and the phenomenon of “under-diagnosis” occurs. Furthermore, not all clinically diagnosed patients have a known genetic defect, suggesting that other genes besides *LDLR*, *APOB* and *PCSK9* may play a role in the alteration of lipid metabolism which remains yet to be discovered.

One drawback of our approach is there was a significant difference noted in our subjects’ age (patients 46.84 SD ±11.2 vs. control 40.00 SD ±9.3), as FH patients recruited were older. Age is acknowledged as risk factor for age-related dyslipidemia. Reason for age-related disruption of lipid homeostasis include the gradual decline in clearance of LDLc with increasing age, the progressively reduced ability to remove cholesterol through conversion to bile acids, the decreased activity of the rate-limiting enzyme in bile acid biosynthesis [44] and progressive decrease in growth hormone secretion with age also causes critical changes in LDLc metabolism. Growth hormone plays an important role in cholesterol homeostasis by either modulating the expression of hepatic *LDLR* [45] or controlling the activity of cholesterol 7alpha-hydroxylase (CYP7A1), a rate-limiting enzyme in the synthesis of bile acid from cholesterol [46]. Presence of FH causal SNPs along with increasing age accelerates the hypercholesterolemia. This bias was beyond our control as our recruitment of subjects was based on volunteered patients who visit UMMC.

Another limitation of our study was that the microarray genotyping platform used in this study only allowed us to study published SNPs. It is possible that rare risk-associated SNPs may be discovered in future using other existing technologies such as sequencing. The authors also recognise that the genotype scores are not true reflection of the biological characteristics of FH as all alleles were given the same statistical weight. Therefore, a model of association of SNPs should be created to calculate the overall genetic risk. These 14 SNPs identified could provide more insights in future studies on screening markers for FH management and warrants further investigation.

## Supporting Information

Figure S1 **Validation by sequencing.**
(TIF)

Table S1 **The Dutch lipid network criteria.**
(DOCX)

Table S2 **HWE values of the significant SNPs.**
(DOC)
