# Supplementary figures and images for: Genetic Polymorphisms in LDLR, APOB, PCSK9 and Other Lipid Related Genes Associated with Familial Hypercholesterolemia in Malaysia
Source: PLoS One. 2013 Apr 8;8(4):e60729. doi: 10.1371/journal.pone.0060729 (PMC3620484; doi:10.1371/journal.pone.0060729)

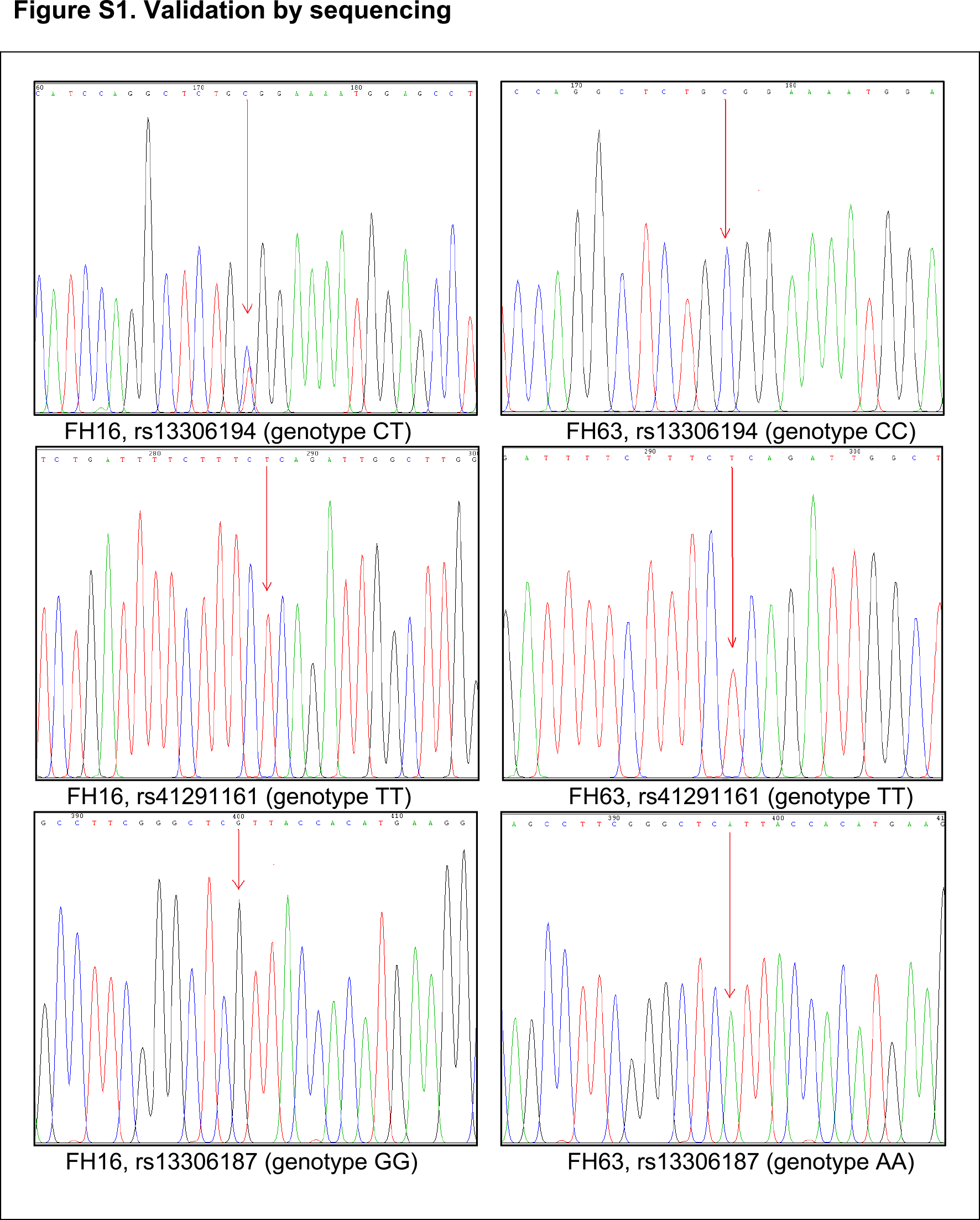

Supplement: Figure S1 — Validation by sequencing. (TIF) [file pone.0060729.s001.tif]
