# Supplementary material for: Genetic Polymorphisms in LDLR, APOB, PCSK9 and Other Lipid Related Genes Associated with Familial Hypercholesterolemia in Malaysia
Source: PLoS One. 2013 Apr 8;8(4):e60729. doi: 10.1371/journal.pone.0060729 (PMC3620484; doi:10.1371/journal.pone.0060729)
Supplement: Table S1 — The Dutch lipid network criteria. (DOCX) [file pone.0060729.s002.docx]

**Table S1. The Dutch Lipid Network Criteria**

| **Criteria** |  |  |
| --- | --- | --- |
| **Family history** |  | **Point** |
| First-degree relative with known premature (men: <55 years; women: <60 years) coronary and vascular disease, or First-degree relative with known LDLC† above the 95th percentile |  | 1 |
| First-degree relative with tendinous xanthomata and/or arcus cornealis, or Children aged less than 18 years with LDLC above the 95th percentile |  | 2 |
| **Clinical history** |  |  |
| Patient with premature (men: <55 years; women: <60 years) coronary artery disease |  | 2 |
| Patient with premature (men: <55 years; women: <60 years) cerebral or peripheral vascular disease |  | 1 |
| Physical examination |  |  |
| Tendinous xanthomata |  | 6 |
| Arcus cornealis prior to age 45 years |  | 4 |
| **Cholesterol levels (mmol/liter)** |  |  |
| LDLC, ≥8.5 |  | 8 |
| LDLC, 6.5–8.4 |  | 5 |
| LDLC, 5.0–6.4 |  | 3 |
| LDLC, 4.0–4.9 |  | 1 |
| **DNA analysis** |  |  |
| Functional mutation in the LDLR gene |  | 8 |

Diagnosis (diagnosis is based on the total number of points obtained)

A “definite” FH† diagnosis requires more than 8 points

A “probable” FH diagnosis requires 6–8 points

A “possible” FH diagnosis requires 3–5 points
