# Supplementary material for: Genetic Polymorphisms in LDLR, APOB, PCSK9 and Other Lipid Related Genes Associated with Familial Hypercholesterolemia in Malaysia
Source: PLoS One. 2013 Apr 8;8(4):e60729. doi: 10.1371/journal.pone.0060729 (PMC3620484; doi:10.1371/journal.pone.0060729)
Supplement: Table S2 — HWE values of the significant SNPs. (DOC) [file pone.0060729.s003.doc]

**Table S2. HWE values of the significant SNPs**

| **rs no.** | **Gene** | **HWE** |
| --- | --- | --- |
| rs2569556 | LDLR | 0.008570067 |
| rs12720772 | APOB | 0.000000001 |
| rs41291161 | APOB | 0.184067159 |
| rs565436 | PCSK9 | 0.310970532 |
| rs13306187 | APOB | 0.49911121 |
| rs13306194 | APOB | 0.422806382 |
| rs12714238 | APOB | 0.21885093 |
| rs12720762 | APOB | 0.197863724 |
| rs12084215 | PCSK9 | 0.471134759 |
| rs28362269 | PCSK9 | 0.046127024 |
| rs57825321 | APOB | 0.00000000 |
| rs12714254 | APOB | 0.000000000 |
| rs2516839 | USF1 | 0.727286435 |
| rs3737787 | USF1 | 0.281418041 |

rs no, NCBI Reference SNP (rs) Number, an identification tag assigned by NCBI to SNPs.
